# Hidden threat in familial Mediterranean fever: subclinical inflammation, oxidative stress and their relationship with vitamin D status

**DOI:** 10.3906/sag-2103-235

**Published:** 2021-09-20

**Authors:** MHD Boshr ALESH, Didem BARLAK KETİ, Ayşenur PAÇ KISAARSLAN, Sabahattin MUHTAROĞLU, Sema Nur TAŞKIN

**Affiliations:** 1 Department of Medical Biochemistry, Faculty of Medicine, Erciyes University, Kayseri, Turkey; 2 Department of Pediatric Rheumatology, Faculty of Medicine, Erciyes University, Kayseri, Turkey

**Keywords:** Familial Mediterranean fever, inflammation, vitamin D, oxidative stress

## Abstract

**Background/aim:**

Vitamin D levels have been investigated in children with familial Mediterranean fever (FMF), but the relationship between vitamin D status and inflammation/oxidative stress indicators could not be clearly demonstrated. This study aimed to investigate the relationship between subclinical inflammation/oxidative stress and vitamin D status in children with FMF during an attack-free period.

**Materials and methods:**

In the cross-sectional study, ninety children with FMF in the attack-free period and 30 healthy children were included. Patients were grouped according to their vitamin D status (< 20, 20–29, and 30–100 ng/mL). The groups were compared in terms of pentraxin 3 (PTX-3), total oxidant status (TOS), and total antioxidant status (TAS). Multivariable linear regression analysis was performed to identify factors associated with vitamin D status.

**Results:**

PTX-3 levels were significantly higher in patients with vitamin D insufficiency (20–29 ng/mL) than in the group with vitamin D sufficient (30–100 ng/mL). Patients with vitamin D deficiency (< 20 ng/mL) had higher TOS. A strong negative correlation was observed between vitamin D levels and TOS (p = 0.003). Subclinical inflammation (PTX-3 ≥ 0.640) and high TOS levels were negatively associated with vitamin D levels.

**Conclusion:**

Subclinical inflammation and oxidative stress were negatively associated with vitamin D levels in patients with FMF during an attack-free period. Sufficient vitamin D levels are important in fighting subclinical inflammation and oxidative stress in children with FMF.

## 1. Introduction

Familial Mediterranean fever (FMF) is an autosomal recessive, characterized by recurrent fever and inflammation of the peritoneum, pleura, or synovium. Patients are generally asymptomatic between attacks, but subclinical inflammation continues in the attack-free period in some patients [1–3].

When the MEFV (Mediterranean FeVer) gene is mutated, the pyrin protein activates caspase-1 and stimulates excessive IL-1β secretion. IL-1β is responsible for inducing the synthesis of acute-phase reactants such as C-reactive protein (CRP), serum amyloid A (SAA), and pentraxin-3 (PTX-3) [1,4]. PTX-3 levels were found to be higher in patients with FMF during the attack and attack-free periods compared to control despite the use of colchicine. Some researchers have suggested that PTX-3 can be an indicator of subclinical inflammation [4–6]. Subclinical inflammation increases the risk of developing complications such as anemia, heart disease, and amyloidosis in patients with FMF. Therefore, the prevention of subclinical inflammation is extremely important. Additionally, investigations have shown that oxidative stress increases in patients with FMF during attack and the attack-free periods [7,8]. In this study, total oxidant status (TOS) and total antioxidant status (TAS) were used to determine oxidative stress. TOS and TAS were detected with practical measurement methods developed by Erel [9,10].

Vitamin D has been reported to have immunomodulatory and anti-inflammatory properties [11,12]. There is a limited number of studies investigating vitamin D levels in children with FMF [13–15]. To our knowledge, although these studies determined that vitamin D levels were lower in children with FMF than in healthy controls, the relationship between vitamin D status and subclinical inflammation/oxidative stress indicators could not be clearly demonstrated. The aim of this study was to examine whether there is a relationship between vitamin D levels and subclinical inflammation/oxidative stress indicators in FMF patients in the attack-free period.

## 2. Materials and methods

### 2.1. Patients and controls

The cross-sectional study was done in the outpatient clinic of Pediatric Rheumatology at Erciyes University Hospital between June 1 and August 31, 2020. The current study was conducted in accordance with the Helsinki Declaration. Ethical approval was obtained from Erciyes University, Faculty of Medicine, Ethics Committee for Clinical Research (2020/93).

Ninety (2–18 age) children with FMF in an attack-free period, diagnosed according to Yalcınkaya et al. [16] and regularly followed and treated in the outpatient clinic of Pediatric Rheumatology were consecutively included in the study, considering inclusion and exclusion criteria. In addition, 30 healthy children matched with the patient group in terms of age and sex were included as the control group.

Patients with metabolic bone disease, malnutrition, chronic renal or hepatic failure, and those with chronic disease accompanying FMF were not included in the study. Moreover, none of the patients were in the attack period and used vitamin D supplements or drugs affecting vitamin D levels.

Ninety children in an attack-free period were grouped by considering their 25(OH) vitamin D levels according to the recommendations published by Holick et al. [17]. These subgroups: group 1 (n = 39): child patients with 25(OH) vitamin D status < 20 ng/mL as deficiency; group 2 (n = 33): child patients with 25(OH) vitamin D status 20–29 ng/mL as insufficient; group 3 (n = 18): child patients with 25(OH) vitamin D status 30–100 ng/mL as sufficient. Also, patients were classified into three groups, patients with M694V mutation, without M694V mutation, and mutation-free. We determined the cut-off value for PTX-3 by ROC analysis. According to this value, the patients were divided into two groups as those with subclinical inflammation (PTX-3 ≥ 0.640) and those without subclinical inflammation (PTX-3 < 0.640) and their vitamin D levels were compared. In addition, patients with FMF were divided into subgroups according to their clinical features and their vitamin D levels were evaluated.

The attack-free period was defined as experiencing no attack for at least 2 weeks after the last attack.

### 2.2. Sample collection

After at least 8 h of fasting, morning blood samples were taken into tubes containing anticoagulant and without anticoagulant. All samples from patients and controls were collected in the same season. Tubes without anticoagulant were centrifuged for 10 min at 2000 g. Separated serum samples were transferred to Eppendorf tubes and stored at −80 °C for 6 months.

### 2.3. All clinical and laboratory assessments

Age, sex, age at diagnosis, duration of disease, FMF symptoms, duration of attacks frequency of attacks, colchicine dose, and its usage duration, genetic mutation analyzes were obtained during the follow-up period from the hospital records. The International Severity Scoring System for FMF (ISSF) criteria (≤ 2 mild, 3–5 intermediate, ≥ 6 severe disease) were used for disease severity scoring [18].

Serum CRP levels were analyzed on Cobas c702 autoanalyzer. The measurement of PTH and 25(OH) D vitamin levels was performed by using the electrochemiluminescence (ECLIA) method on Cobas e802 immunoassay analyzer (Roche Diagnostics, Mannheim, Germany). Erythrocyte sedimentation rate (ESR) was measured in tubes containing anticoagulant on Vision analyzer (China).

Total oxidant status (TOS) and total antioxidant status (TAS) were analyzed using the spectrophotometric kit (Rel Assay Diagnostics, Gaziantep/Turkey). ELISA was utilized for PTX-3 measurement (YL Biotech, China).

### 2.4. Statistical analysis

“IBM SPSS Statistics 23” statistical package program was used to evaluate the data. The compliance of the data to normal distribution was evaluated using the histogram and Q-Q plot and Shapiro–Wilk test. Summary statistics of numerical variables with and without normal distribution were given as mean ± standard deviation and median (25% –75% percentile) values, respectively. Statistical comparisons were done using the Student t*-*test and ANOVA for normally distributed data. Mann-Whitney U and Kruskal-Wallis tests were used for non-normally distributed data. Post-hoc analyses were done by using Bonferroni correction. Chi-square test was used for comparison of categorical variables. Correlation analysis was done by Pearson or Spearman tests. ROC analysis was applied to determine the cut-off value for PTX-3. Multivariable linear regression analysis was performed to determine the factors associated with serum vitamin D levels. Independent variables were included in the model via blockwise entry. Vitamin D level was defined as the dependent variable. Sex was included in the model as an independent variable (Model 1). Seasonal difference in the frequency of attacks, TOS, and PTX3 were added (Model 2). The model fit was checked with a scatter plot of the predicted value versus residuals, the correlation matrix, tolerance, or variance inflation factor (VIF). Adjusted R-square was evaluated of the model. The power analysis for calculation of the sample size was done using G^*^Power v.3.1.9.2. The required sample size was 30 participants in each group (control and patient) for serum PTX3 and TOS (power = 0.80 at α = 0.05). In all statistical comparisons, the significance level was accepted as p < 0.05.

## 3. Results

When the age means and sex distribution of the patient and control groups (11.95 ± 3.95 and 11.55 ± 4.26, p = 0.636, boy (50% and 56.6%); girl (50% and 43.3%), p = 0.527, respectively) were compared, no significant difference was found.

### 3.1. Clinical Features

The distribution of 90 children with FMF according to clinical characteristics was shown in Table 1. Twenty-four (26.7%) patients had the seasonal difference in the frequency of attacks and had more attacks in winter. Seventy-one (79%) and nineteen (21%) children were evaluated as mild and moderate, respectively, according to the disease severity score.

No statistically significant difference was found between vitamin D and the frequency of the attacks (p = 0.911) and duration of the attacks (p = 0.172) and disease severity score (p = 0.621), but vitamin D levels were lower in patients with the seasonal difference in the frequency of attacks (p = 0.014) (Table 2). Additionally, vitamin D levels were higher in boys [25.9 (19.95–31.95)] than in the girls [17.2 (11.65–23.75)] (p < 0.001).

### 3.2. Genetic Findings

The distribution of the patients according to the M694V gene mutations was shown in Table 3. Thirty-one (64.4%) patients had M694V gene mutations. Although vitamin D levels did not differ between groups with and without M694 gene mutation, vitamin D levels were found to be significantly higher in the group mutation-free than in the patients with gene mutations (p = 0.020) as shown in Table 3.

### 3.3. Biochemical Analysis Findings

As seen in Table 4, PTX-3 and TOS levels were higher in the patient group compared to the control, while the TAS level was low. No statistically significant differences were found between the patient and control groups in terms of vitamin D and PTH levels.

In Table 5, ESR and TAS values did not differ among the vitamin D subgroups (vitamin D deficiency, vitamin D insufficient, and vitamin D sufficient) (p = 0.314 and p = 0.185, respectively). Group 1 (vitamin D deficiency) and group 2 (vitamin D insufficient) had lower CRP levels when compared to group 3 (vitamin D sufficient) (p = 0.041).

The percentages of FMF patients with vitamin D sufficient, insufficient, and deficiency were determined as 20, 36.7, and 43.3, respectively. Thirty percent of the control group had vitamin D deficiency.

**Figure 1:**
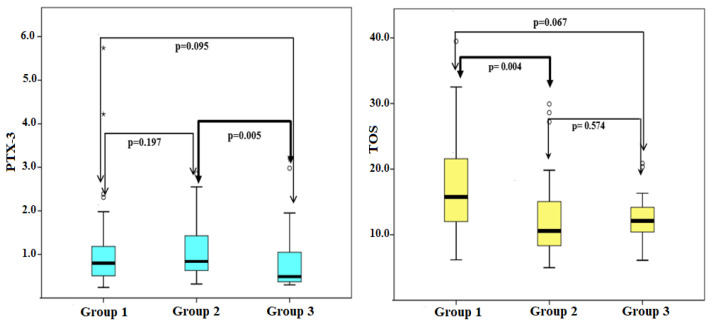
Comparison of PTX-3 and TOS values in patient groups.

As shown in Figure 1, PTX-3 values differed significantly among the subgroups (p = 0.028). In the group 3, PTX-3 levels [0.49 (0.37–1.09)] were lower than in the group 2 [0.84 (0.63–1.51)] (p = 0.008). PTX-3 levels did not show difference between sexes (p = 0.738). TOS values differed significantly between groups separated according to vitamin D status (p = 0.011). The TOS values were higher in the group 1 [15.74 (12.00–22.78)] than in the group 2 [10.56 (8.08–15.57)]. This difference was statistically significant (p = 0.004).

**Figure 2:**
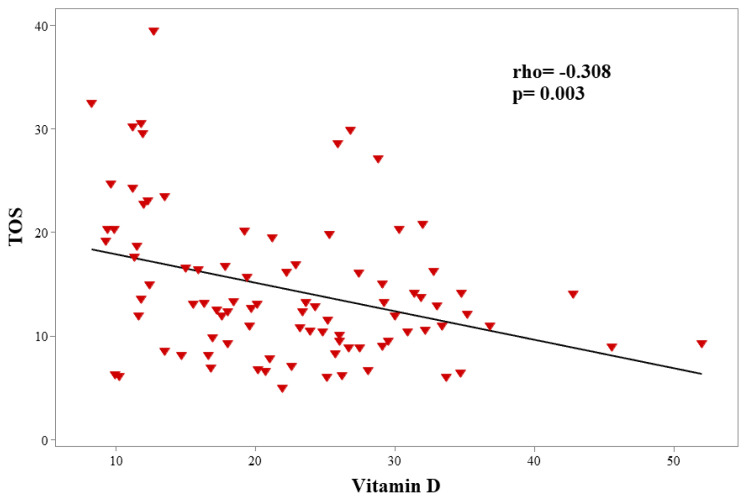
Correlation of vitamin D with TOS in patients with FMF.

A significant negative correlation was observed between vitamin D and TOS (p = 0.003 rho = –0.308) in patients with FMF in Figure 2. However, no statistically significant relation was detected between vitamin D level and other clinical, laboratory variables in children with FMF.

**Figure 3:**
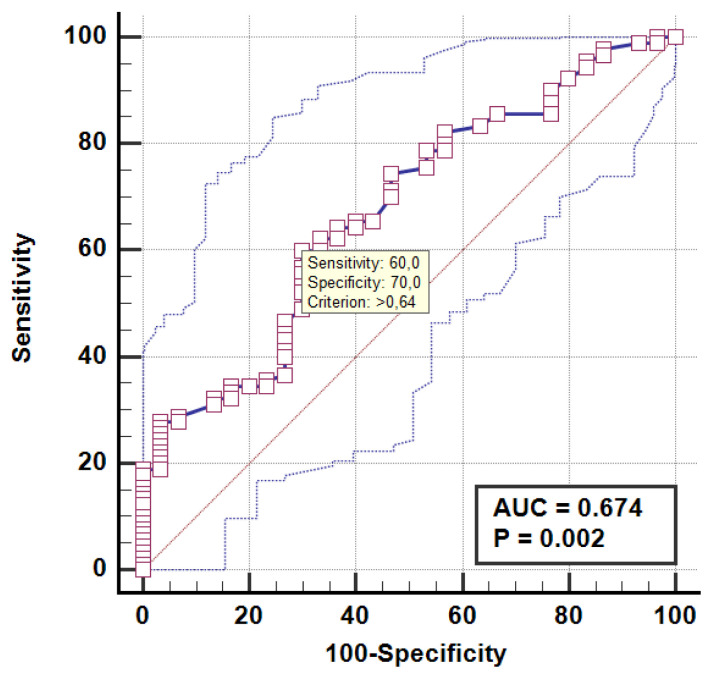
ROC for PTX-3.

ROC analysis was applied to determine the cut-off value for PTX-3 in Figure 3. In the multivariable linear regression analyses as shown in Table 6, it was found that girl sex, subclinical inflammation (PTX-3 ≥ 0.640), high TOS levels and exposure to more attacks in winter were negatively associated with vitamin D levels in patients with FMF. While the first model (sex) helped to explain 17.7% of the variance, the final model helped to explain 32.4% of the variance when PTX3, TOS, and seasonal difference in frequency of the attacks were added.

## 4. Discussion

Subclinical inflammation generates a hidden threat to the development of FMF complications such as amyloidosis in attack-free intervals [3]. In the present study, the difference in subclinical inflammation (PTX-3), and oxidative stress indicators (TOS) between the patient and control groups indicates the presence of subclinical inflammation in the attack-free period.

Vitamin D levels have been investigated in children with FMF, but the relationship between vitamin D status and inflammation/oxidative stress indicators could not be clearly demonstrated.

Studies have not clarified whether vitamin D deficiency is a consequence or cause of inflammatory disease. Some researchers hypothesized that low vitamin D is the consequence of a chronic inflammatory process caused by persistent infection [19].

It has been suggested that low vitamin D levels in patients with FMF may induce subclinical inflammation since vitamin D has an immunomodulatory effect on Th cells and affects cytokine production [20].

One of the main results of this study is that subclinical inflammation is one of the factors associated with vitamin D levels. Multivariable linear regression analysis revealed that subclinical inflammation was negatively associated with vitamin D levels in patients with FMF.

Vitamin D both decreases the production of pro-inflammatory mediators such as IL-1, IL-6, TNF-α and increases the production of IL 10, an anti-inflammatory cytokine. Therefore, it has anti-inflammatory activity on macrophages, dendritic cells, monocytes, and NKs. It has been determined that the regulation of 1α-hydroxylase in immune system cells is significantly different from kidneys and that 1α-hydroxylase is stimulated by cytokines such as TNF and IFN [11,21–23]. Therefore, we believe that sufficient vitamin D levels are important in combating subclinical inflammation.

In previous studies, vitamin D levels in pediatric FMF patients [13–15,24,25] were lower than controls; it has been reported that it is higher in boys than in girls [26]. Kozan et al. [27], found to be similar vitamin D levels between patients with FMF and controls. Vitamin D deficiency and insufficiency were found in 26% and 62% of the children with FMF, respectively [20]. In another study, 83.3% of the patients had vitamin D deficiency during the attack-free period [24]. In the present study, vitamin D levels were deficient in 43.3% of FMF patients and 30% of controls. However, the similarity of vitamin D levels with the control may be due to the fact that the study was planned in the summer months.

Vitamin D levels were sufficient in 20% of patients with FMF. Additionally, as similar to the other studies [14,15,26], we found that vitamin D levels were higher in boys than in girls.

Erten et al. [26] demonstrated that inflammatory indicators (ESR and fibrinogen) were correlated with lower vitamin D levels in adult patients with FMF, although no relationship was found between the vitamin D levels and clinical characteristics of child patients or acute phase reactants in other studies [13–15,24]. Different results obtained in the study of Erten et al. [26] may be associated with the age and the higher CRP levels (14.3 ± 34.3 mg/L) of the patients. Similarly, there was no significant relationship between vitamin D levels and disease severity score [14,20]. These results were consistent with the present study.

Studies showed that vitamin D levels are similar in FMF patients with different MEFV gene mutations. The most prevalent mutation was detected in the M694V gene [15,26]. In the present study, M694V was the most common gene mutation and vitamin D levels were similar in FMF patients with and without M694V gene mutation. Patients with gene mutations had lower vitamin D levels than those gene mutation-free. Further studies are needed to clarify the relationship between genotype and vitamin D.

It was known that acute phase reactants were higher in the attack period than in the attack-free period. In a study, ESR was similar between the attack-free patient and the healthy groups [5]. However, another study found that ESR was significantly higher in the attack-free group compared to the control group [4]. In addition to studies reporting that CRP levels do not differ between patient group with attack-free period and control group [6,8,28], there are also studies showing the opposite [5]. Nevertheless, ESR and CRP were also within normal levels in the asymptomatic period. Therefore, we think that a more sensitive indicator could be useful instead of CRP and ESR in reflecting subclinical inflammation.

Moreover, CRP is an acute phase reactant synthesized particularly in the liver by the action of proinflammatory cytokines. PTX-3 is produced as a result of by stimulation of toll-like receptors (TLRs) and pro-inflammatory cytokines (IL-1β and TNF-α) and released from peripheral mononuclear cells [29,30]. Therefore PTX-3 may be more specific than CRP in reflecting subclinical inflammation. It has been proposed that vitamin D has immunomodulatory functions via modulation of TLRs and leads to decrease expression of TRL2 in autoimmune and pro-inflammatory based diseases [31]. Therefore, the relationship between vitamin D and inflammation can be better reflected with PTX-3. In the present study, PTX3 levels were low in FMF patients with sufficient vitamin D status.

Gok et al. [4] determined that the PTX-3 level was higher in young adults in the attack-free period compared to the control, and PTX-3 had a sensitivity of 90% at a threshold value of 0.696 ng/mL. In the present study, we divided the patients into two groups according to cut-off value (0.640) for PTX-3, we found that vitamin D levels were significantly lower in the patient group with subclinical inflammation.

Zhang et all. [32] reported that serum vitamin D levels should be kept above 30 ng/mL to obtain sufficient anti-inflammatory effects. In the present study, PTX-3 levels were higher in groups with vitamin D insufficient compared to the group with sufficient vitamin D. Its levels of at least 30 ng/mL could be effective in preventing subclinical inflammation.

Reactive oxygen species (ROS) produced in activated inflammatory cells by cytokines cause oxidative stress in patients with FMF [3,10]. The low serum paraoxonase (PON1) levels and increased lipid peroxidation during the attack-free period of FMF lead to the continuity of oxidative stress [25,33]. Although Savran et al. [34] detected higher TOS in adult patients with FMF compared to control, TAS results were found to be similar. In another study, while TAS during the attack and attack-free periods was lower, TOS was found to be higher than in the control. Moreover, higher TOS values were detected during the attack compared to the attack-free period [35].

In the present study, the group with vitamin D deficiency (< 20 ng/mL) also constituted the group with the highest TOS values. Vitamin D levels above 20 ng/mL caused a significant decrease in TOS. TAS did not show a significant difference between the groups separated according to vitamin D status. In patients with FMF, the negative relationship between vitamin D and TOS was interpreted as vitamin D levels can be effective in reducing reactive oxygen radicals.

The absence of a diseased control group is one of the main limitations of the study. Thus, we could not determine PTX3 in the diseased control group. Vitamin D levels were not evaluated during attack and attack-free periods of the disease. The number of patients was low in groups formed according to vitamin D levels. Additionally, serum amyloid A levels of patients could not be measured.

In conclusion, the present study showed that subclinical inflammation and high TOS levels were negatively associated with vitamin D levels. Sufficient vitamin D levels are important in fighting subclinical inflammation and oxidative stress in patients with FMF. Therefore, routine vitamin D measurement and vitamin D supplementation can be beneficial for patients with vitamin D deficiency. We believe that the findings obtained from this study will gain more value by conducting randomized controlled studies.

## Figures and Tables

**Table 1:** Distribution of patients with FMF according to clinical characteristics.

Symptoms	n (%)
Abdominal pain	73 (81)
Fever	72 (80)
Arthralgia/arthritis	51 (56)
Chest pain	19 (2)
Rash	2 (2.2)
**Attack duration in the last year**	
≤ 2 days	52 (57.8)
≥3 days	38 (42.2)
**Frequency of the attack**	
≤ 2 / years	41 (45.6)
3-4 / years	4 (4.4)
≥ 5 / years	9 (10)
No attack in the last year	36 (40)
**Seasonal difference in attack frequency**	
No	66 (73.3)
Yes-winter	24 (26.7)
**Disease severity score**	
≤ 2 (mild)	71 (79)
3-5(moderate)	19 (21)
≥ 6 (severe)	-
**Treatment**	
Colchicine	80 (88.9)
Biologic drugs	10 (11.1)
**Duration of treatment (years)**	
Colchicine	5.19 ± 3.67
Biologic drugs	1.92 ± 1.23
**Dose of colchicine (mg/day)**	
0.5–1 mg	75 (83.3)
1.5–2 mg	15 (16.7)

**Table 2:** Comparison of vitamin D levels of patients according to their clinical characteristics

Variables	n	Vitamin D	p
**Duration of the attacks in the last year**			0.172
≤ 2 days	52	21.05 (12.15–26.75)	
≥3 days	38	24.15 (17.2–29.70)	
**Frequency of the attacks**			0.911
≤ 2 / years	41	23.20 (16.70–28.95)	
>2 / years	13	21.00 (17.40–30.60)	
**Disease severity score**			0.621
≤ 2 (mild)	71	22.20 (15.6–27.47)	
3-5 (moderate)	19	21.0 (12.0–30.67)	
**Seasonal difference in frequency of the attacks**			0.014
No	66	24.55 (16.90–29.20)	
Yes-winter	24	17.30 (12.15–22.05)	

Data were presented as median (25% - 75% percentile)

**Table 3:** The comparison of vitamin D levels according to M694V gene mutation.

Mutation	n	%	Vitamin D	p
**Patients with M694V mutation**	58	(64.4)	21.30 ± 8.15^a^	0.020
**Patients without M694V mutation**	25	(27.7)	22. 03 ± 8.92^b^	
**Patients with mutation-free**	7	(7.9)	31.20 ± 11.86^a^,^b^	

Data were presented as n (%), mean ± standard deviation.

a Patients with M694V mutation vs mutation-free; p = 0.016.

b Patients without M694V mutation vs mutation-free; p = 0.046.

**Table 4:** Comparison of demographic data and some parameters of the patients with FMF and controls.

Variables	Patients with FMF (n = 90)	Controls (n = 30)	p
**25 (OH) D (ng/mL)**	22.05 (14.93–28.88)	22.70 (17.90–26.35)	0.509
**PTH (pg/mL)**	27.85 (21.48–36.30)	27.85 (17.98–36.03)	0.490
**CRP (mg/L)**	0.63 (0.31–2.25)	0.31 (0.17–0.64)	< 0.001
**PTX-3 (ng/mL)**	0.8 (0.5–1.33)	0.5 (0.4–0.93)	0.004
**TAS** (μmol/L)	0.97 (0.9–1.06)	1.03 (0.97–1.16)	0.007
**TOS**(μmol/L)	12.92 (9.30–17.91)	8.24 (6.04–10.42)	< 0.001

Data were presented as median (25%–75% percentile).

**Table 5:** Comparison of CRP, ESR and TAS according to vitamin D levels.

Variables	Group 1 (n=39) Vitamin D insufficient	Group 2 (n=33) Vitamin D deficiency	Group 3 (n=18) Vitamin D sufficient	P
**CRP (mg/L)**	0.53 (0.31–1.38)	0.62 (0.30–1.62)	3.72 (0.5–5.28)	0.041
**ESR**	6.2 (4.25–11.5)	4.57 (2.8–11.25)	6.0 (3.71–12.0)	0.314
**TAS** (μmol/L)	0.97 (0.93–1.13)	0.96 (0.86–1.08)	0.96 (0.90–1.0)	0.185

Data were presented as median (25%–75% percentile).

**Table 6:** Multivariable linear regression analysis

Model		Unstandardized Coefficients		Standardized Coefficients	p	95% Confidence Interval for B		Collinearity Statistics	
		Beta	Std. Error	Beta		Lower bound	Upper bound	Tolerance	VIF
1	(constant)	18.430	1.214		<0.001	16.018	20.841		
	sex	7.695	1.716	0.431	<0.001	4.284	11.106	1.000	1.000
2	(constant)	26.963	2.247		<0.001	22.495	31.430		
	sex	6.774	1.576	0.380	<0.001	3.641	9.908	0.974	1.027
	TOS	−0.334	0.112	−0.264	0.004	−0.558	−0.111	0.968	1.033
	Seasonal difference in attack frequency	−4.290	1.767	−0.213	0.017	−7.804	−0.776	0.990	1.010
	PTX3	−3.456	1.598	−0.190	0.033	−6.634	−0.278	0.986	1.014

Dependent variable: Vitamin D

Model 1: Independent variable; se

Model 2: Independent variables; sex, TOS, Seasonal difference in frequency of the attacks (no or yes-winter) and

PTX3 (subclinical inflammation no or yes according to cut-off 0.640 for PTX-3).

Model 1: Adjusted R^2^ = 17.7%

Model 2: Adjusted R^2^ = 32.4%

Group 1: vitamin D deficiency, group 2: vitamin D insufficient, group 3: vitamin D sufficient.
